# Origin of the Amphibian Chytrid Fungus

**DOI:** 10.3201/eid1012.030804

**Published:** 2004-12

**Authors:** Ché Weldon, Louis H. du Preez, Alex D. Hyatt, Reinhold Muller, Rick Speare

**Affiliations:** * North-West University, Potchefstroom, South Africa;; † CSIRO, Geelong, Australia;; ‡ James Cook University, Townsville, Australia

**Keywords:** Chytridiomycota, chytridiomycosis, Batrachochytrium dendrobatidis, amphibian chytrid, amphibian declines, disease, epidemiology, origin, prevalence, southern Africa, Xenopus, amphibians, research

## Abstract

The sudden appearance of chytridiomycosis, the cause of amphibian deaths and population declines in several continents, suggests that its etiologic agent, the amphibian chytrid *Batrachochytrium dendrobatidis*, was introduced into the affected regions. However, the origin of this virulent pathogen is unknown. A survey was conducted of 697 archived specimens of 3 species of *Xenopus* collected from 1879 to 1999 in southern Africa in which the histologic features of the interdigital webbing were analyzed. The earliest case of chytridiomycosis found was in a *Xenopus laevis* frog in 1938, and overall prevalence was 2.7%. The prevalence showed no significant differences between species, regions, season, or time period. Chytridiomycosis was a stable endemic infection in southern Africa for 23 years before any positive specimen was found outside Africa. We propose that Africa is the origin of the amphibian chytrid and that the international trade in *X. laevis* that began in the mid-1930s was the means of dissemination.

## Abstract 2

Histologic evidence indicates southern Africa as the origin of the amphibian chytrid fungus.

One of the biggest threats facing amphibian species and population survival worldwide is the disease chytridiomycosis, caused by the chytrid fungus, *Batrachochytrium dendrobatidis* (*1*,*2*). Chytridiomycosis was proposed as the cause of death in frog populations in the rain forests of Australia and Panama and was associated with the decline of frog populations in Ecuador, Venezuela, New Zealand, and Spain (*3*–*6*). Evidence for a countrywide decline in frog populations in South Africa is lacking (*7*), and local declines of several species have been ascribed to two main threats, habitat destruction and pollution (*8*). Chytridiomycosis is known in South Africa from infections in *Xenopus laevis*, *Afrana fuscigula*, and *Strongylopus grayii* (*9*–*11*). Through surveys of extant and archived specimens, *Batrachochytrium* has been found in every continent that has amphibians, except Asia (*6*,*9*,*12*,*13*). Since *B. dendrobatidis* has been recognized as an emerging pathogen, whose spread is facilitated by the international and intranational movement of amphibians (*1*), identifying its origin will be useful.

Some emerging infectious diseases arise when pathogens that have been localized to a single host or small geographic region go beyond previous boundaries (*14*). If *B. dendrobatidis* emerged in this fashion, we hypothesize that the source would meet the following criteria: 1) the hosts would show minimal or no apparent clinical effects, 2) the site would be the place of the earliest known global occurrence, 3) the date of this occurrence would precede any amphibian declines in pristine areas (i.e., late 1970s), 4) the prevalence in the source host or hosts would be stable over time, 5) no geographic spreading pattern would be observed over time in the region, 6) a feasible means of global dissemination of *Batrachochytrium* from the region of origin would be identified, and 7) *B. dendrobatidis* would show a greater genetic variation in the host region than in more recently invaded regions.

*B. dendrobatidis* is common in African frogs from Ghana, Kenya, South Africa, and Western Africa (*12*,*15*) and declines in frog populations are poorly documented in Africa (*7*,*16*). These factors, combined with the global trade in *X. laevis* and *X. tropicalis*, prompted us to investigate the likelihood that Africa was the origin of *Batrachochytrium* and that the trade in *Xenopus* spp. played a key role in its global dissemination. Within the *Xenopus* genus, *X. laevis* is distributed over the greatest area in sub-Saharan Africa. *X. laevis* occupies most bodies of water in savannah habitats from the Cape of Good Hope to Nigeria and Sudan (*17*,*18*).

We report the earliest case of the amphibian chytrid found in any amphibian and present epidemiologic evidence to support the hypothesis that *B. dendrobatidis* originated in Africa. In this article, chytridiomycosis refers to infection of amphibians by *B. dendrobatidis*.

## Materials and Methods

A retrospective survey was conducted on archived specimens of the genus *Xenopus* housed in five southern Africa institutions, Bayworld (Port Elizabeth), Natal Museum (Pietermaritzburg), National Museum (Bloemfontein), South African Museum (Cape Town), and Transvaal Museum (Pretoria). Specimens in these museums had been collected for archiving by a large number of persons for various purposes and had not been selected for a systematic survey of amphibian disease. Specimens were collected mainly from South Africa, Lesotho, and Swaziland. A piece (3 x 3 mm) of the interdigital webbing was removed from one hind foot of each specimen of *X. gilli*, *X. muelleri*, and *X. laevis*. Tissue was prepared for histologic examination with routine techniques (*19*). Sections were cut at 6 μm and stained with hematoxylin and eosin. Chytridiomycosis was diagnosed by using described criteria (*20*). Sections from the two specimens diagnosed as having chytridiomycosis with hematoxylin and eosin before 1971 (one collected in 1938, the other in 1943) were confirmed with the more specific immunoperoxidase test (*21*) to increase the confidence of the diagnosis. Measurements of sporangia were performed with a calibrated eyepiece and expressed as mean ± standard deviation (SD). Histologic slides were examined "blind," without reference to dates that the frogs were collected, to decrease any opportunity for bias in diagnosis.

Exact versions of chi-square tests were used to analyze bivariate associations between chytridiomycosis prevalence and host species, region in South Africa (southwestern, eastern, and central), and season. Bivariate time trends of prevalences were analyzed by exact chi-square tests for trend. Multivariate logistic regression models were applied to assess potential confounding effect of species, region, and season on the time trend of chytridiomycosis prevalence. Confidence intervals (CI) were calculated by using exact binomial probabilities. Longitudinal and latitudinal historical patterns of spread were analyzed with linear regression models.

## Results

**Figure 1:**
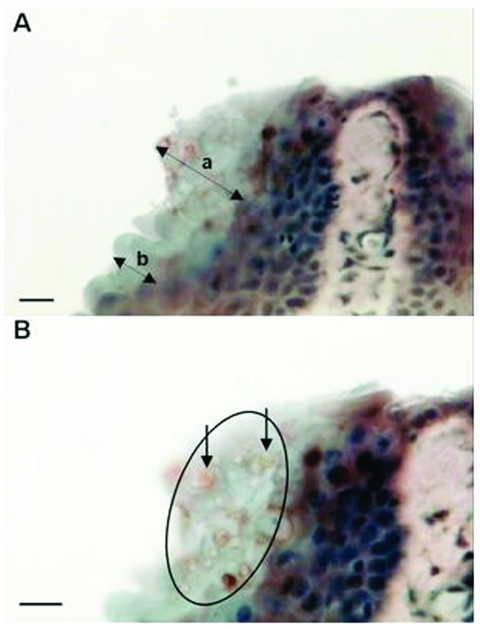
Micrographs of immunoperoxidase stained sections through the interdigital webbing of *Xenopus gilli*, showing the morphologic features and size of zoosporangia consistent with *Batrachochytrium dendrobatidis*. A) Arrow a indicates localized hyperplastic epidermal response; arrow b indicates an uninfected region of the epidermis. B) Arrows indicate two zoosporangia with internal septa. Circle indicates location of the infection in the stratum corneum. Bar, 10 μm.

Zoosporangia with a diameter (mean ± SD) of 5.2 ± 0.72 μm (maximum 6 μm) were seen in the stratum corneum of the digital webbing of infected frogs (Figure 1). Most sporangia were empty spherical structures, but occasional sporangia were observed with developing stages, septa, or discharge papillae. The structures stained brown (indicating positivity) in the immunoperoxidase test with the specific anti-*Batrachochytrium* antibody (Figure 1). Lesions usually associated with chytridiomycosis, including hyperplasia of the epidermis and hyperkeratosis of the stratum corneum, were mild and localized to areas of infection.

**Figure 2:**
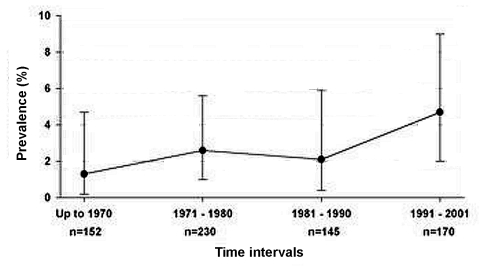
Historical time-trend of chytridiomycosis prevalence in southern Africa. No significant change was shown in the prevalence over time (p = 0.22, 95% confidence interval).

Overall, chytridiomycosis prevalence from the survey was 2.7% (19 positives out of 697 specimens) and did not differ significantly across species (p = 0.7; Table 1). The earliest date for a chytridiomycosis-positive specimen was 1938 in an *X. laevis* collected from the Western Cape coastal lowland. This specimen is housed in the South African Museum, Cape Town (SAMZR 18927). The next earliest positive specimen detected was an *X. gilli* from 1943 (specimen number NMB 112, National Museum, Bloemfontein). The distribution of dates specimens were collected was greatly skewed to the latter half of the 20th century (Table 2). The breakdown for the time interval 1871–1940 is presented in order (decade, number of frogs infected/number of frogs examined) as follows: 1871–1880, 0/1; 1881–1890, 0/0; 1891–1900, 0/6; 1901–1910, 0/6; 1911–1920, 0/4; 1921–1930, 0/2; 1931–1940, 1/37. No statistically significant change of chytridiomycosis prevalence occurred over the decades since the 1940s (p = 0.36), or when the broader interval of pre-1971 is used as the baseline for the calculations (p = 0.22; Figure 2). No evidence for any trend in prevalence over time could be found using multivariate modeling where the odds ratios for the time intervals were adjusted for the potential confounders of species, season, and region. The multivariate odds ratios in these models were not significant and very similar to the bivariate findings, which indicate no confounding effects. The prevalence of chytridiomycosis in South Africa showed no significant change over time after 1940. No significant change of the geographic distribution of chytridiomycosis was detected after 1973. By 1973 the distribution of chytridiomycosis, as proved by positive specimens, covered already the area from 27° to 34° latitude and 18.25° to 32.5° longitude. This finding implies that positive specimens were detected from all regions of southern Africa by 1973. Infected frogs were found in 5 of the 9 provinces in South Africa, including the Western Cape (5 of 171), Northern Cape (2 of 22), Free State (6 of 141), Kwazulu-Natal (3 of 152), and Eastern Cape (1 of 137), as well as in Swaziland (2 of 42). Prevalence of *B. dendrobatidis* did not differ (p = 0.24) between the designated three broader regions with prevalences of 3.0% in the southwest, 3.8% in the central region, and 1.5% in the eastern region. Overall, the seasons (wet versus dry) when the specimens were collected were not significantly associated with prevalence (p = 0.22). Only in the eastern region, was a significantly higher prevalence found in the wet season than the dry season.

**Table 1:** Prevalence of chytridiomycosis in archived *Xenopus* spp. from southern Africa^a^

Species	No. examined	% positive (95% CI)	Earliest positive detected	Country
*Xenopus laevis*	583	2.6 (1.5–4.2)	1938	South Africa
*X. meulleri*	53	3.8 (0.5–13.0)	1991	Swaziland
*X. gilli*	61	3.3 (0.4–11.4)	1943	South Africa
Total	697	2.7		

^a^p = 0.7; CI, confidence interval.

**Table 2:** Prevalence of chytridiomycosis in archived *Xenopus*, by time intervals^a^

Time interval	No. examined	No. positives	% positive (95% CI)
1871–1940	56	1	1.8 (0.0–9.6)
1941–1950	16	1	6.3 (0.2–30.2)
1951–1960	63	0	0.0 (0.0–5.7)
1961–1970	17	0	0.0 (0.0–19.5)
1971–1980	230	6	2.6 (1.0–5.6)
1981–1990	145	3	2.1 (0.4–5.9)
1991–2001	170	8	4.7 (2.0–9.0)
Total	697	19	2.7 (1.7–4.2)

^a^p = 0.36; CI, confidence interval.

## Discussion

**Figure 3:**
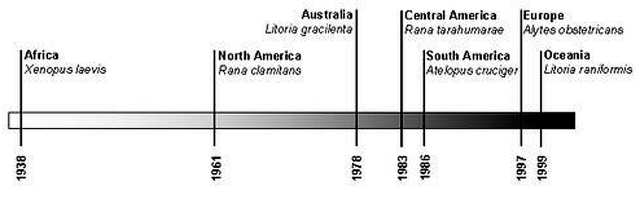
Time bar indicating when chytridiomycosis first appeared in the major centers of occurrence in relation to each other. Following a 23-year interruption in occurrences after the *Xenopus laevis* infection in 1938, records outside Africa appear with increasing frequency up until the present; North America (*22*), Australia (*2**,**23*), South America (*5*), Central America (*24*), Europe (*6*), Oceania (New Zealand) (*25*).

Our study has extended the date for the earliest case of chytridiomycosis in wild amphibians by 23 years. The next earliest case outside South Africa was found in *Rana clamitans* from Saint-Pierre-de-Wakefield, Québec, Canada, in 1961 (*22*). After the case in Canada, the earliest cases from other countries follow sequentially over a period of 38 years from 1961 to 1999 (Figure 3).

*X. laevis* in the wild does not show clinical signs, nor has it experienced any sudden die-offs. Moreover, only subclinical chytrid infections have been observed among captive colonies of *X. laevis* (*26*,*27*). A frog of a related species, *X. tropicalis*, died in captivity from chytridiomycosis, it was suspected of having contracted the fungus from *X. laevis* (*27*). An ideal host for transmission of chytridiomycosis through international translocation would be a species of amphibian that does not become diseased or die from the infection; hence, *X. laevis* could take on the role of a natural carrier.

The sudden appearance of chytridiomycosis can best be explained by the hypothesis that *B. dendrobatidis* was recently introduced into new regions and subsequently infected novel host species (*1*). Dispersal of *B. dendrobatidis* between countries is most likely by the global transportation of amphibians (*1*,*2*,*23*,*28*,*29*). The World Organization for Animal Health has recently placed amphibian chytridiomycosis on the Wildlife Diseases List in recognition of this risk. If Africa is the source of *B. dendrobatidis*, a feasible route of dissemination by infected amphibians needs to be identified. Some members of the family *Pipidae* have been exported, in particular *Hymenochirus curtipes* and *X. laevis*, to North America and Europe (*30*).

In terms of a most likely candidate for spread from Africa, the number of frogs and geographic dissemination favor *X. laevis*. Soon after discovery of the pregnancy assay for humans in 1934 (*30*), enormous quantities of the species were caught in the wild in southern Africa and exported around the world. The pregnancy assay is based on the principle that ovulation in *X. laevis* is induced by injection with urine from pregnant women because of high levels of gonadotropic hormones in the urine (*31*,*32*). *X. laevis* was selected as the most suitable amphibian for investigating the mechanism of the mating reflex because of the relative ease with which the animal can be maintained in captivity (*33*). For 34 years, the trade in *X. laevis* in South Africa was controlled by the then Cape of Good Hope Inland Fisheries Department (Western Cape Nature Conservation Board) at the Jonkershoek Fish Hatchery. As an indication of the numbers involved in this trade, 10,866 frogs were distributed in 1949, of which 3,803 (35%) were exported, and of the 20,942 frogs distributed in 1970, a total of 4,950 (24%) were shipped abroad (*34*,*35*). After the introduction of nonbiologic pregnancy tests, *X. laevis* became important as a model for the scientific study of immunity and later embryology and molecular biology. *X. laevis* could have carried the disease globally, particularly if the prevalence was similar to that seen in wild-caught *X. laevis* today. In the importing country, escaped frogs, the water they lived in (*36*), or both, could have come into contact with local amphibian species, and subsequent transmission of the disease could have followed. The establishment of feral populations of *X. laevis* in Ascension Island, the United Kingdom, the United States, and Chile in 1944, 1962, the 1960s, and 1985 (*37*), respectively, show that transmission could have become ongoing if these feral populations were infected.

Although we have demonstrated that *B. dendrobatidis* was in southern Africa since 1938, our studies provide no indication regarding whether this region was the original source within Africa. *B. dendrobatidis* has been found in wild frogs in Kenya and in frogs (*X. tropicalis* and *X. laevis*) wild-caught in Western Africa and detected after importation into the United States (*12*,*26*,*27*,*38*), which indicates that *B. dendrobatidis* is widely disseminated in Africa. *Xenopus* consists of 17 species that are found in sub-Saharan Africa, with a varying degree of sympatry between species (*17*). The overlap in the distribution and, in some cases, the sharing of habitats could facilitate transmission of *B. dendrobatidis* between these species. This finding would imply that chytridiomycosis could have originated elsewhere in Africa and spread within multiple host-region combinations. More detailed historical studies of archived African amphibians may indicate whether *B. dendrobatidis* was originally present in a small area of Africa from which it emerged to occupy large areas of the continent. Until the deficit in distribution data and comparative genetic studies is remedied, locating the source of the origin of *B. dendrobatidis* within Africa remains speculative. The relationship appears to have coevolved within an anuran host, and the opportunity to disseminate across the globe existed for *B. dendrobatidis* in southern Africa.

If *X. laevis* did carry *B. dendrobatidis* out of Africa as we propose, other amphibian species subsequently could have distributed it between and within countries. The American bullfrog, *Rana catesbeiana*, has been proposed as an important vector, mainly through international trade as a food item, but also within countries as populations established for the food trade escape and spread (*29*). The earliest current record for the occurrence of chytridiomycosis in *R. catesbeiana* is 1978 in South Carolina (*38*), 40 years after the first record in southern Africa, but details on the intensity of the search for chytridiomycosis in archived bullfrogs are not available. The transmission of chytridiomycosis globally may involve a series of key steps: 1) occurrence of *B. dendrobatidis* in an amphibian vector in southern Africa that is relatively resistant to disease (*X. laevis*), 2) sudden rise in 1935 of export trade in this vector because of technologic advances (*Xenopus* pregnancy test), 3) escape of the pathogen from the exported *Xenopus* to establish new foci in other countries (possibly expedited in some countries by establishment of feral populations of *X. laevis*), 4) transmission into other vector amphibians (food and pet trade), and 5) further transmission to other countries along different trade routes in key amphibian vectors that move in high numbers and become established in commercial populations and closely interact with wild frogs, which likely leads to feral populations (food frogs *R. catesbeiana*). Spread through native amphibian populations with epidemic disease in some species could have occurred at any point after *B. dendrobatidis* entered a naïve native species.

We have provided epidemiologic evidence that Africa is the origin of the amphibian chytrid fungus. Support for six of the seven criteria proposed for the source of *B. dendrobatidis* has been demonstrated: 1) the major host (*X. laevis*) shows minimal or no apparent clinical effects, 2) site of the earliest global occurrence (1938), 3) this date precedes any amphibian declines in pristine areas, 4) the prevalence in the source host or hosts (*Xenopus* spp.) has been stable over time, 5) no geographic spreading pattern could be observed over time, and 6) a feasible means of global dissemination exists via the international trade in wild-caught *X. laevis*, which commenced in 1935 and continues today. Criterion 7, greater genetic diversity of *B. dendrobatidis* at the source, has not been investigated. A low level of genetic variation was shown for 35 strains of *B. dendrobatidis* and suggested that *B. dendrobatidis* was a recently emerged clone (*39*). The strains had been collected in North America, Australia, Panama, and Africa from wild and captive amphibians. Three strains isolated from captive *X. tropicalis* in United States had been imported from Ghana. Although these showed no significant differences from the U.S. strains (*39*), their assignment to Africa assumes no cross-infection had occurred within the importing facility. Future work on the genetic diversity of *B. dendrobatidis* in Africa compared with strains from regions outside Africa will add weight to the hypothesis if greater genetic diversity is found in African strains.
